# Shared peptide binding of HLA Class I and II alleles associate with cutaneous nevirapine hypersensitivity and identify novel risk alleles

**DOI:** 10.1038/s41598-017-08876-0

**Published:** 2017-08-17

**Authors:** Rebecca Pavlos, Elizabeth J. McKinnon, David A. Ostrov, Bjoern Peters, Soren Buus, David Koelle, Abha Chopra, Ryan Schutte, Craig Rive, Alec Redwood, Susana Restrepo, Austin Bracey, Thomas Kaever, Paisley Myers, Ellen Speers, Stacy A. Malaker, Jeffrey Shabanowitz, Yuan Jing, Silvana Gaudieri, Donald F. Hunt, Mary Carrington, David W. Haas, Simon Mallal, Elizabeth J. Phillips

**Affiliations:** 1 Institute for Immunology and Infectious Diseases, Murdoch University, Murdoch, WA 6150 Australia; 2 University of Florida College of Medicine, Gainesville, FL 32610 USA; 3 La Jolla Institute for Allergy and Immunology, La Jolla, CA 92037 USA; 4 Department of Immunology and Microbiology, University of Copenhagen, Copenhagen, DK-2200 Denmark; 5 Department of Medicine, University of Washington, Seattle, WA 98195 USA; 6 Department of Global Health, University of Washington, Seattle, WA 98195 USA; 7 Vaccine and Infectious Diseases Division, Fred Hutchinson Cancer Research Center, Seattle, WA 98109-1024 USA; 8 Department of Laboratory Medicine, University of Washington, Seattle, WA 98195 USA; 9 Benaroya Research Institute, Seattle, WA 98195 USA; 10 Departments of Chemistry and Pathology, University of Virginia, Charlottesville, VA 222904 USA; 11 Boehringer Ingelheim Pharmaceuticals Inc., Ridgefield, CT 06877 USA; 12 School of Anatomy, Physiology and Human Biology, University of Western Australia, Crawley, WA 6009 Australia; 13 Vanderbilt University School of Medicine, Nashville, TN 37232 USA; 14 Cancer and Inflammation Program, Laboratory of Experimental Immunology, Leidos Biomedical Research Inc., Nashville, TN 37232 USA; 15 Frederick National Laboratory for Cancer Research, Frederick, MD 21702-1201 USA; 16 Ragon Institute of MGH, MIT and Harvard, Cambridge, MA 02139 USA; 17 Meharry Medical College, Nashville, TN 37208 USA

## Abstract

Genes of the human leukocyte antigen (HLA) system encode cell-surface proteins involved in regulation of immune responses, and the way drugs interact with the HLA peptide binding groove is important in the immunopathogenesis of T-cell mediated drug hypersensitivity syndromes. Nevirapine (NVP), is an HIV-1 antiretroviral with treatment-limiting hypersensitivity reactions (HSRs) associated with multiple class I and II HLA alleles. Here we utilize a novel analytical approach to explore these multi-allelic associations by systematically examining HLA molecules for similarities in peptide binding specificities and binding pocket structure. We demonstrate that primary predisposition to cutaneous NVP HSR, seen across ancestral groups, can be attributed to a cluster of HLA-C alleles sharing a common binding groove F pocket with HLA-C*04:01. An independent association with a group of class II alleles which share the HLA-DRB1-P4 pocket is also observed. In contrast, NVP HSR protection is afforded by a cluster of HLA-B alleles defined by a characteristic peptide binding groove B pocket. The results suggest drug-specific interactions within the antigen binding cleft can be shared across HLA molecules with similar binding pockets. We thereby provide an explanation for multiple HLA associations with cutaneous NVP HSR and advance insight into its pathogenic mechanisms.

## Introduction

Adverse drug reactions are associated with considerable global morbidity and mortality and pose a substantial challenge in drug development and implementation. A subset of these reactions are T-cell mediated and associate with particular class I and/or class II human leukocyte antigen (*HLA*) alleles, which govern presentation of peptides for recognition by the T-cell receptor (TCR).

The peptide binding grooves of both class I and class II HLA molecules are formed by a β-sheet floor consisting of eight anti-parallel β-sheets, packed against two anti-parallel α-helices forming a channel^1^. In class I molecules (HLA-A, -B, and -C) the binding groove is divided into six pockets, A-F, which are defined by specific polymorphic amino acid residues that determine their topography and functionality^2–6^. These class I HLA molecules typically bind peptides 8–11 amino acids in length. Structures of peptide/HLA complexes show that conserved hydrogen bonds are formed between HLA side chains and the peptide backbone of the nine core amino acids within the bound peptide^7^. Additional HLA allele specific interactions are formed between the peptide side chains and structural pockets in the antigen binding cleft. Compared to class I, the class II HLA-DRB1 molecules bind longer peptides of variable length (i.e. 12–15 amino acids). The most polymorphic HLA-DRB1 elements are the structural pockets that accommodate peptide positions 1 (P1), P4, P6, P7 and P9^7^.

The allelic specificity of the HLA peptide binding groove in the pathogenesis of T cell mediated drug hypersensitivity is exemplified by the well characterized abacavir hypersensitivity syndrome which occurs both *in vivo* and *in vitro* only in association with HLA-B*57:01, and not with related B17 serotype alleles such as HLA-B*57:02/3 and HLA-B*58:01. It is well established that patients carrying these related alleles tolerate abacavir and *in vitro* functional assays are negative. This illustrates the importance of allele-specific sites within the HLA peptide binding groove, where single amino acid changes seen between risk and control alleles can alter the chemistry of HLA-drug interaction. Abacavir binds directly to a unique combination of polymorphic residues within the F pocket of the HLA binding groove present only in HLA-B*57:01 and not in other B17 serotype alleles^8, 9^. This results in presentation of self-peptides not previously exposed to patient T cells as neoantigens^8–10^. Dependence on the structure of the antigen binding groove for determining HLA allelic risk has also been demonstrated for other drug hypersensitivity syndromes^11–15^.

Nevirapine (NVP) is antiretroviral active against HIV-1, which is generally well tolerated without central nervous system, metabolic or renal toxicities. However, treatment-limiting drug-induced hypersensitivity reactions (HSR) affect approximately 5% of patients who initiate nevirapine and this has impacted use of the drug globally. These HSRs are also noted in patients treated with NVP for HIV post-exposure prophylaxis^16, 17^. NVP hypersensitivity encompasses different clinical phenotypes with cutaneous, hepatic or systemic symptoms^18^. The different HSR phenotypes are associated with both shared and specific class I and class II HLA alleles, which have variable distribution and risk across ethnic groups^19–21^. Cutaneous reactions range in severity from mild rash through to severe diseases with high morbidity and mortality such as Stevens Johnson Syndrome/Toxic Epidermal Necrolysis (SJS/TEN) and drug reaction with eosinophilia and systemic symptoms (DRESS), which is characterized by a combination of fever, rash and/or hepatitis and/or eosinophilia^19^. The HLA alleles most often associated with cutaneous manifestations of NVP HSR are HLA-C*04, commonly carried across ethnicities, as well as HLA-B*35 in Asians and Caucasian patients^19, 21–24^.

In this work we consider how HLA allelic groupings based on similarities in peptide binding specificity and structure of the HLA binding groove may explain observed diversity of HLA associations with the severe cutaneous phenotype of NVP HSR (grade 3 or 4 rash). Validated supertypes, which group alleles based on peptide binding data and pocket chemistry^4, 5, 25^, are examined, together with class I and II allele clusters defined by similarities in pocket structure of the peptide-binding groove^4, 5, 25^. This approach has identified key HLA loci specific positions within the binding groove associated with cutaneous NVP HSR and several novel risk and protective HLA alleles for the development of the syndrome.

## Results

### Cutaneous NVP HSR associates with HLA-C alleles having similar peptide binding properties and F pocket structure as HLA-C*04:01

**Figure 1:**
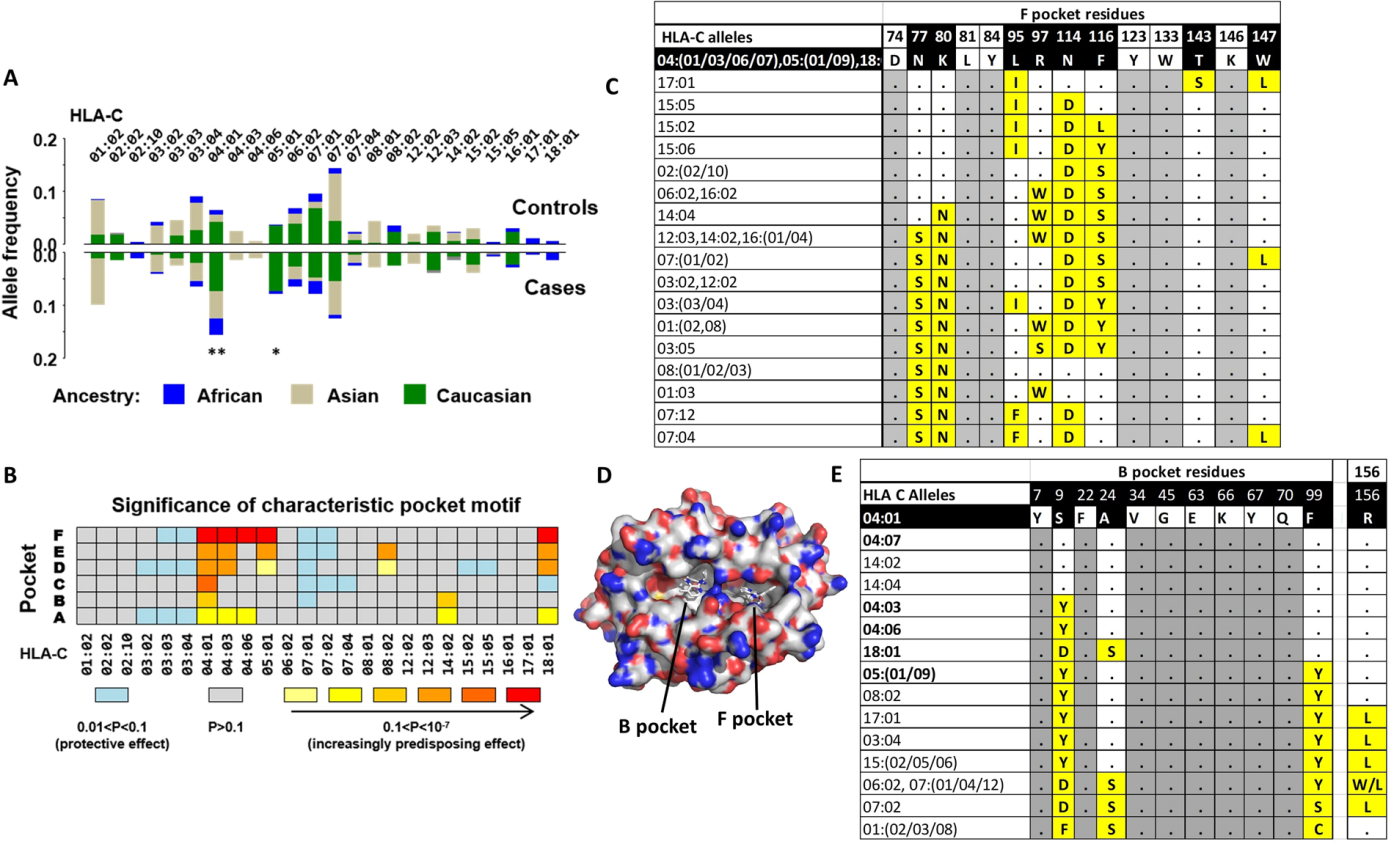
HLA-C alleles with shared F pocket and binding properties associate with cutaneous NVP HSR. Summaries of *HLA-C* alleles prevalent in this cohort (≥5 carriers). (**A**) Relative allele frequencies amongst cases (N = 151) and controls (N = 413) according to ancestral group. **Carriage of HLA-C*04:01 vs non-carriage: Odds ratio 3.06 (adjusted for ethnicity), *P* < 0.0001; *HLA-C*05:01: Odds ratio = 2.67, *P* = 0.002. (**B**) Heatmap illustrating impact on development of cutaneous NVP HSR for each *HLA-C* allele according to the relative significance of its characteristic motif across the HLA binding pockets A-F. Protective motifs are denoted by blue, and predisposing motifs range in color from yellow (weak effect) through to red (strongest effect). (**C**) Alignment of HLA-C F pocket sequences. Yellow highlighted positions show amino acids that are variable amongst the cohort alleles and conserved within the *HLA-C* risk group for cutaneous NVP HSR. (**D**) Molecular docking model showing preferred locations of NVP bound to the peptide binding groove of HLA-C*04:01 within the B or F pocket as determined by positional scanning analysis. (**E**) Alignment of representative HLA-C B pocket sequences and position 156. Yellow highlighted positions show amino acids that are variable amongst the cohort alleles and conserved within the *HLA-C* risk group for cutaneous NVP HSR. NVP HSR risk alleles from this analysis with a common F pocket are shown in bold font. All other HLA-C alleles from the cohort with n > 5 are not shown and carry the HLA-B pocket common to risk alleles except at 9-Y(Tyr), 99-Y(Tyr), and 156 L/W/Q (Leu/Trp/Gln).

Four digit HLA typing was available for 151 cases and 413 controls. In single allele logistic regression analyses HLA-C*04:01 was the only allele for which a consistent, significant predisposing relationship for cutaneous manifestations of NVP HSR was observed across all ancestral groups (Odds ratio (OR) = 3.06 and *P* = 0.0001 in whole cohort analysis, (Fig. 1A); Asian: OR = 5.49, *P* = 0.0001; Caucasian: OR = 2.08, *P* = 0.02; and African: OR = 3.84, *P* = 0.04). However, analyses specific to ancestral groups also revealed several other *HLA-C* allelic associations indicative of HSR predisposition, namely HLA-C*05:01 in Caucasians (versus non-HLA-C*05:01 carriers: OR = 2.84, *P* = 0.002) and HLA-C*18:01 in patients with African ancestry (versus non-HLA-C*18:01 carriers: OR = 2.67, *P* = 0.2; vs non-HLA-C*04:01/-C*18:01 carriers: OR = 4.71, *P* = 0.06).

Similarities between binding specificities for the identified *HLA-C* risk alleles (HLA-C*04:01, -*05:01 and -*18:01) were examined with *MHCcluster* (which groups HLA molecules according to their peptide-binding specificity^26, 27^) and according to their characteristic motif across pockets (A-F) of the HLA-C peptide-binding groove^3–6^. Respective consideration of pocket composition characterised a subset of HLA-C risk alleles^3–6^. For every pocket, the characteristic HLA-C*04:01 motif demonstrated greatest impact on development of cutaneous NVP HSR (Fig. 1B), with the greatest significance attributable to the F pocket^4^, where commonality of the residues Asp74-Asn77-Lys80-Leu81-Tyr84-Leu95-Arg97-Asn114-Phe116-Tyr123-Trp133-Thr143-Lys146-Trp147 grouped risk alleles HLA-C*05:01 and HLA-C*18:01 with HLA-C*04:01 in a cluster that also included HLA-C*-04:03 and -*04:06* (Fig. 1C). Other HLA-C alleles with similarities in peptide binding preference predicted by *MHCcluster* differed at several F pocket positions (HLA-C*17:01, -C*08:02, -C*14:02, -C*07:01/*02*/04, -C*06:02) (Fig. 1C, Figure S1). Characterization of other HLA binding pockets A-E by key amino acid residues failed to group the primary *HLA-C* risk HSR alleles together, or conversely included additional alleles that weakened the associated effect. Moreover, the heightened risk of cutaneous NVP HSR conferred by the HLA-C*04:01 cluster could not simply be attributed to greater surface expression levels for the risk alleles. A modest univariable association with HLA-C expression imputed from published MFI coefficients^28–30^ was abrogated in an analysis that jointly considered carriage of an allele belonging to the predisposing *HLA-C* cluster (expression level: *P* > 0.2; risk HLA-C allele: *P* = 0.0001), although we note relative size of observed risk effects reflect the ordering of imputed expression levels^28–30^ (MFI expression units: C*05:01 = 154 < C*04:01 = 199 < C*18:01 = 239; multivariable OR[95% CI]: C*05:01/09 = 2.2[1.2–3.9] < C*04:01/03/06 = 2.5[1.6–3.9] < C*18:01 = 2.6[0.6–11.1]).

Since *HLA-C* risk alleles share F pocket residues we hypothesized that a common direct interaction between the F pocket of the antigen-binding cleft and drug/peptide may drive a common predisposition to cutaneous NVP HSR. Molecular docking and positional scanning was utilised to predict potential interactions between NVP with the antigen binding cleft using the crystal structure of HLA-C*04:01 ^31^ and the most likely positions for NVP to bind to HLA-C*04:01 is either within the B pocket, close to position 99 of the binding groove or within the F pocket (Fig. 1D, Table S1). This agrees with an independent analysis by *Carr et al*.^32^ Not all identified HLA-C risk alleles carry Phe99, the exception being HLA-C*05:01 which carries Tyr99 and other B pocket residues in common with non-risk alleles (Fig. 1E). However, position Arg156 of the binding groove was also shared by risk alleles (Fig. 1E, Figure S2) and this position is important in HLA-C*04:01 crystal structure with peptide (QY**D**DAVYKL), providing stability to the **D** at P3 of the bound peptide, enabling P3 to act as an alternative N terminal anchor residue^31^. Therefore, the observed association of F pocket residues with cutaneous NVP HSR are consistent with modelling data that suggest this may be a preferred docking site for NVP within the HLA-C peptide binding groove. In addition, the position Arg156 shared by all risk alleles may be important in providing stability to the bound peptide in the presence of NVP.

### Secondary associations with cutaneous NVP HSR attributable to HLA class I binding pocket structure

**Figure 2:**
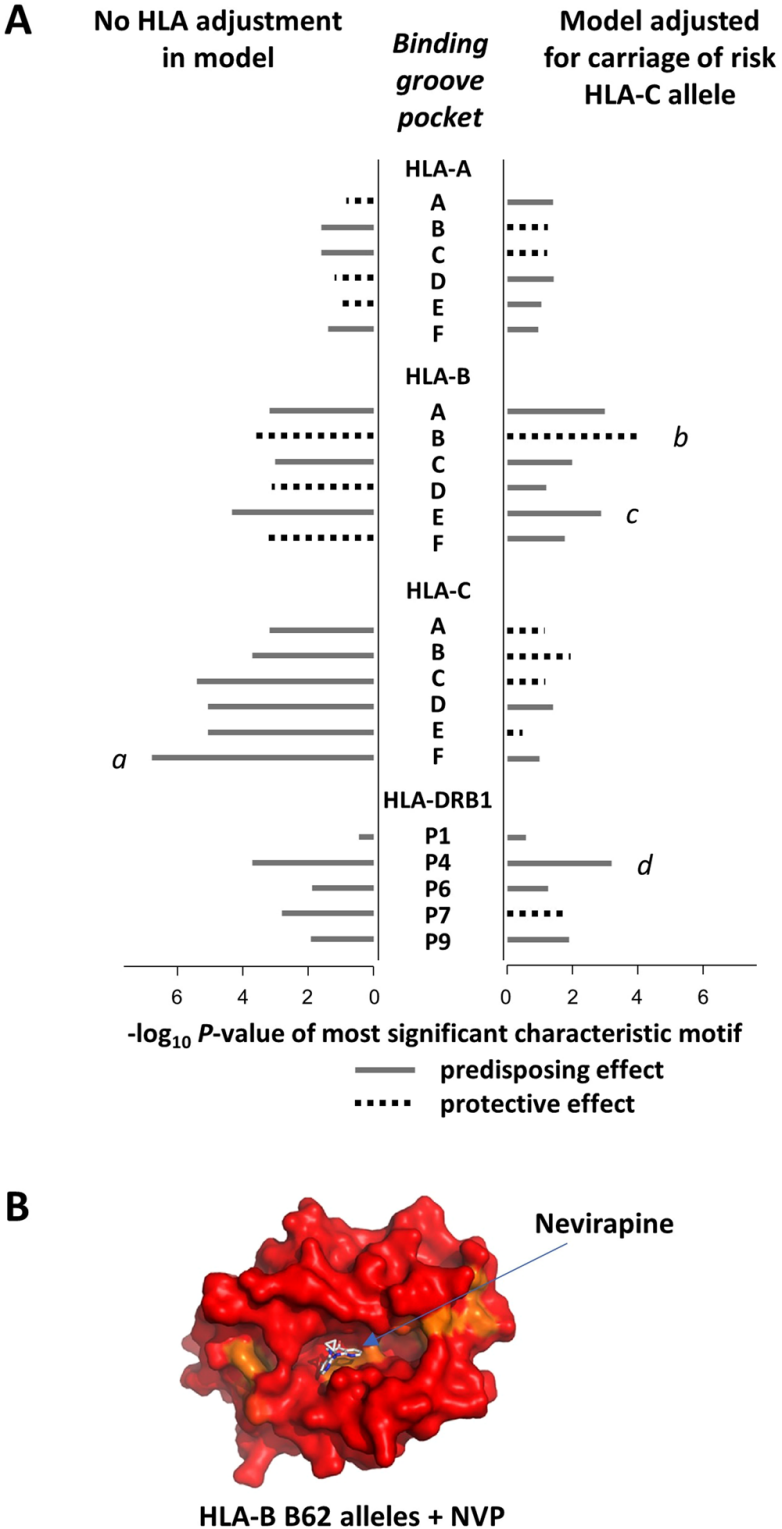
Principal associations with cutaneous NVP HSR across each pocket of the peptide binding groove for *HLA-A*, *-B*,*-C* and -*DRB1*. (**A**) Results show the *P*-value for the characteristic motif having greatest association with cutaneous NVP HSR in ethnicity-adjusted logistic regression analyses with and without additional adjustment for co-carriage of the *HLA-C* risk F pocket. Alleles sharing the noted characteristic motifs include: (*a*) primary risk alleles HLA-C*04:01/03/06,-C*05:01,-C*1801; (*b*) protective HLA-B B62 alleles HLA-B*15:01/12/24/25/27/32/35 and -B*52:01; (*c*) risk allele HLA-B*35:05; (*d*) *HLA-DRB1* risk cluster -DRB1*01:01/02/03 and -DRB1*04:04/05/08/10. (**B**) Molecular docking predictions of NVP binding to protective HLA-B*15:01. The structure of HLA-B*15:01 (PDB 1XR8) is colored according to sequence similarity with HLA-B B62 supertype protective alleles. Blosum62 similarity values are: blue, 40–50, cyan, 50–60, green, 60–70, yellow, 70–80, orange 80–90, and red 90–100. Molecular docking predicts that NVP interacts with a structural B pocket largely shared by HLA-B B62 supertype molecules, as indicated with a blue line.

Having established that *HLA-C* alleles sharing the HLA-C*04:01 F pocket^4^ have a primary predisposing impact on development of cutaneous NVP HSR, we next sought to elucidate the role of secondary HLA class I and class II effects. We similarly considered peptide binding properties and structure of pockets A-F of the class I loci HLA-A, -B and -C and pockets P1, P4, P6, P7 and P9 of class II HLA-DRB1 within the peptide binding groove^2–7, 33^. P-value plots of the most significant characteristic motif associated with each pocket demonstrate that little effect could be attributed to HLA-A, and the most prominent secondary effect is protection associated with the HLA-B B pocket, which is independent of HLA-C risk (OR = 0.18, p = 0.0001 and OR = 0.20, p = 0.0003 for models with and without adjustment for the primary HLA-C cluster) (Fig. 2A). The B pocket^4, 5^ motif Tyr7-Tyr9-Ala24-Val34-(Met/Thr)45-Glu63-Ile66-Ser67-Asn70-(Tyr/Phe)99 is characteristic of a cluster of HLA-B B62 supertype alleles: HLA-B*15:(*01*/12/24/25/27/35) and HLA-B*52:01 (Figure S3 and Table S2), consistent with a previously reported protective HLA-B*15 association with cutaneous NVP reactions observed in an Indian population^34^. However, in this dataset the strength of the protection is diminished when the HLA B62 supertype as a whole is considered or if HLA-B*52:01 is excluded (Table 1). It appears that the protective property of this HLA B62 sub-group is driven by the common B pocket structure of the binding groove. Other HLA B62 supertype alleles (HLA-B*15:02, HLA-B*46:01) differ from protective alleles at one or more key B pocket positions; Glu63, Ile66, Ser67 or Asn70 (Table S2). Crystallography studies of HLA-B*15:01 structure show a hydrophobic B pocket^35^, and molecular docking of NVP against the protective *HLA-B* alleles provides support for the binding of NVP to this hydrophobic B pocket of the HLA-B peptide binding groove (Fig. 2B).

**Table 1:** HLA-B supertype and *MHCcluster* (2 digit) group associations with NVP HSR rash.

	Supertype	Adjusted for race only			Adjusted for race and predisposing C*0401 cluster		
		OR	[95% CI]	P	OR	[95% CI]	P
	B07	1.75	[1.19–2.58]	0.004	1.64	[1.11–2.44]	0.01
	B08	0.98	[0.48–2.00]	>0.9	1.39	[0.67–2.92]	0.4
	B27	1.00	[0.63–1.58]	>0.9	1.07	[0.67–1.72]	0.8
	B44	1.02	[0.69–1.51]	0.9	0.92	[0.61–1.38]	0.7
	B58	0.68	[0.39–1.18]	0.2	0.68	[0.39–1.19]	0.2
	B62	0.47	[0.28–0.79]	0.004	0.49	[0.29–0.83]	0.009
**Principal supertype**	**MHCCluster**	**OR**	**[95% CI]**	**P**	**OR**	**[95% CI]**	**P**
B07	B*07	0.53	[0.26–1.06]	0.07	0.69	[0.34–1.41]	0.3
B07	B*35; *53	2.09	[1.28–3.41]	0.003	1.25	[0.73–2.16]	0.4
B07	B*51; *54; *55; *56	1.66	[1.05–2.63]	0.03	2.09	[1.29–3.39]	0.003
B08	B*08	0.98	[0.48–2.00]	>0.9	1.39	[0.67–2.92]	0.4
B27	B*14	0.65	[0.27–1.57]	0.3	0.64	[0.26–1.57]	0.3
B27	B*38; *39	1.50	[0.86–2.61]	0.2	1.82	[1.02–3.22]	0.04
B58	B*57; *58	0.79	[0.45–1.39]	0.4	0.78	[0.44–1.39]	0.4
B62	B*46	0.98	[0.49–1.94]	>0.9	1.12	[0.56–2.25]	0.7
B62	B*15	0.43	[0.25–0.74]	0.002	0.40	[0.23–0.70]	0.001
B44	B*37; *40; *45; *50	0.60	[0.36–1.01]	0.05	0.67	[0.39–1.13]	0.1
B44	B*18; *44	1.54	[0.97–2.43]	0.07	1.16	[0.72–1.89]	0.5
B62	B*52	0.19	[0.03–1.48]	0.1	0.22	[0.03–1.73]	0.2
Unclassified	B*13	1.15	[0.64–2.07]	0.6	1.12	[0.62–2.05]	0.7
B27	B*27	0.69	[0.27–1.73]	0.4	0.68	[0.27–1.74]	0.4

The most prominent HLA-B motif associated with risk for cutaneous NVP HSR was the E pocket sequence Ser97-Asp114-Trp147-Val152-Leu156 uniquely carried by HLA-B*35:05 (Fig. 2A, Table S3). Notably, all other HLA-B*35 alleles lack the Ser97 residue and significant predisposition did not extend to the HLA-B*35 allelic group considered as a whole (Table 1). In this cohort, haplotypic HLA-C*04:01 is carried with HLA-B*35:05 in 9 of 10 Southeast Asian cutaneous NVP HSR cases so it is possible that the alleles may act synergistically. Other secondary HLA-B risk alleles were identified by analysis of supertypes in combination with *MHCcluster* designated peptide binding specificities after adjusting for the effect of the HLA-C*04:01 risk allele group (Figure S3, Table 1). These included HLA-B*51/-B*54/-B*55/-B*56 from the supertype B07 and HLA-B*38/-B*39 from supertype B27. While there was no single common HLA binding pocket motif that characterised the *HLA-B* B07 and B27 supertype risk alleles, the HLA-B*38/-B*39 group was characterised by the E/F pocket sequence Leu95-Arg97-Asn114-(Phe/Lys)116-Val152-Leu156 and the B07 HLA-B risk alleles by the sequence Trp95-Thr97-Asn114-(Phe/Leu/Tyr)116-(Val/Glu)152-Leu156 (Table S3).

### Secondary associations with cutaneous NVP HSR attributable to HLA class II, P4 binding pocket

From *MHCcluster* (Figure S4) and binding groove analyses of the *HLA-DRB1* alleles within the cohort we identified a cluster including both supertype HLA-DR1 and -DR4 alleles (HLA-DRB1*01:(*01*/02/03) and -DRB1*04*:*(*04*/05/08/10), respectively) that exhibited a secondary association with cutaneous NVP HSR (Fig. 2A). HLA-DRB1*01:01 and HLA–DRB1*01:02 have previously been associated with hepatic hypersensitivity to NVP^36, 37^. The identified risk alleles for cutaneous NVP HSR share the sequence (Phe/His)13-Gln70-Arg71-Ala74-Tyr78 over residues forming the P4 pocket (Table S4). In contrast, the predominantly Caucasian alleles HLA-DRB1*04:01 and -DRB1*04:15 differed from the risk group only at position β71 within the P4 pocket, and yet appeared to be protective in this ancestral group (2 cases, 26 controls, p = 0.01). This suggests that β71, which is key in dictating the nature of the peptide amino acid that is accommodated in the P4 pocket of the antigen binding groove, is of particular importance in NVP HSR. The β71 position within the P4 pocket of HLA-DRB1 has previously been implicated in the pathology of several autoimmune diseases including Graves disease, type I diabetes and rheumatoid arthritis^38–42^.

Elution studies utilising L2 cell lines that stably express the NVP HSR risk allele HLA–DRB1*01:01 were conducted to assess if presence of the drug may affect the repertoire of peptides that bind to HLA-DRB1*01:01. For HLA-DRB1*01:01, 605 peptides were eluted in the untreated cells and 359 from the NVP treated cells. Peptides in the unique +/− NVP elution sets (untreated: N = 305; treated: N = 64) were of similar length (mean (sd) treated = 14.6 (2.6); untreated = 15.0 (2.8)), and the comparison of their core 9mers as predicted using *NETMHCIIpan version* 2.*2* showed very similar distributions for anchoring amino acids at P1 and P9 (Figure S5). Apparent small differences in the frequency of some amino acids at P4 and P6 did not reach statistical significance with adjustment for multiple comparisons. Finally, all peptides from the NVP treated sample (>12 mer) were examined for differences in binding affinity in the presence and absence of the drug in functional assays. No difference in binding to HLA-DRB1*01:01 was observed.

Molecular modelling and docking scores were used to investigate whether NVP is likely to be accommodated by the HLA-DRB1*01*:01* antigen binding cleft in the presence of peptide utilising the sequence logo for peptides eluted in the absence and presence of NVP (Figures S5 and S6). Both models predicted weak binding scores (−6.1 kcal/mol) and suggested that NVP is unlikely to bind directly to HLA-DRB1*01:01 in the presence of peptide (Figure S6). In all, this data provides little support for NVP having a significant effect on the binding of peptides to HLA-DRB1*01:01.

### Overall model and risk assessment for cutaneous NVP HSR

**Figure 3:**
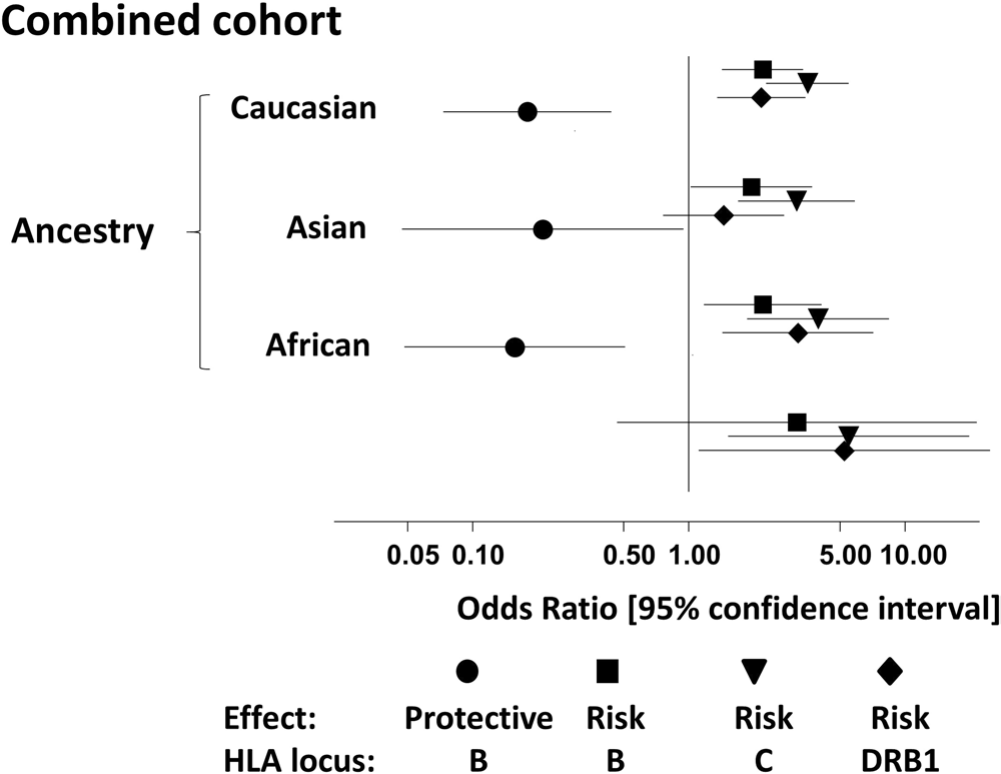
Relative effects of predisposing and protective HLA clusters on cutaneous NVP HSR risk. Odds ratios have been estimated from multivariate logistic regression modelling with adjustment for ethnicity.

**Figure 4:**
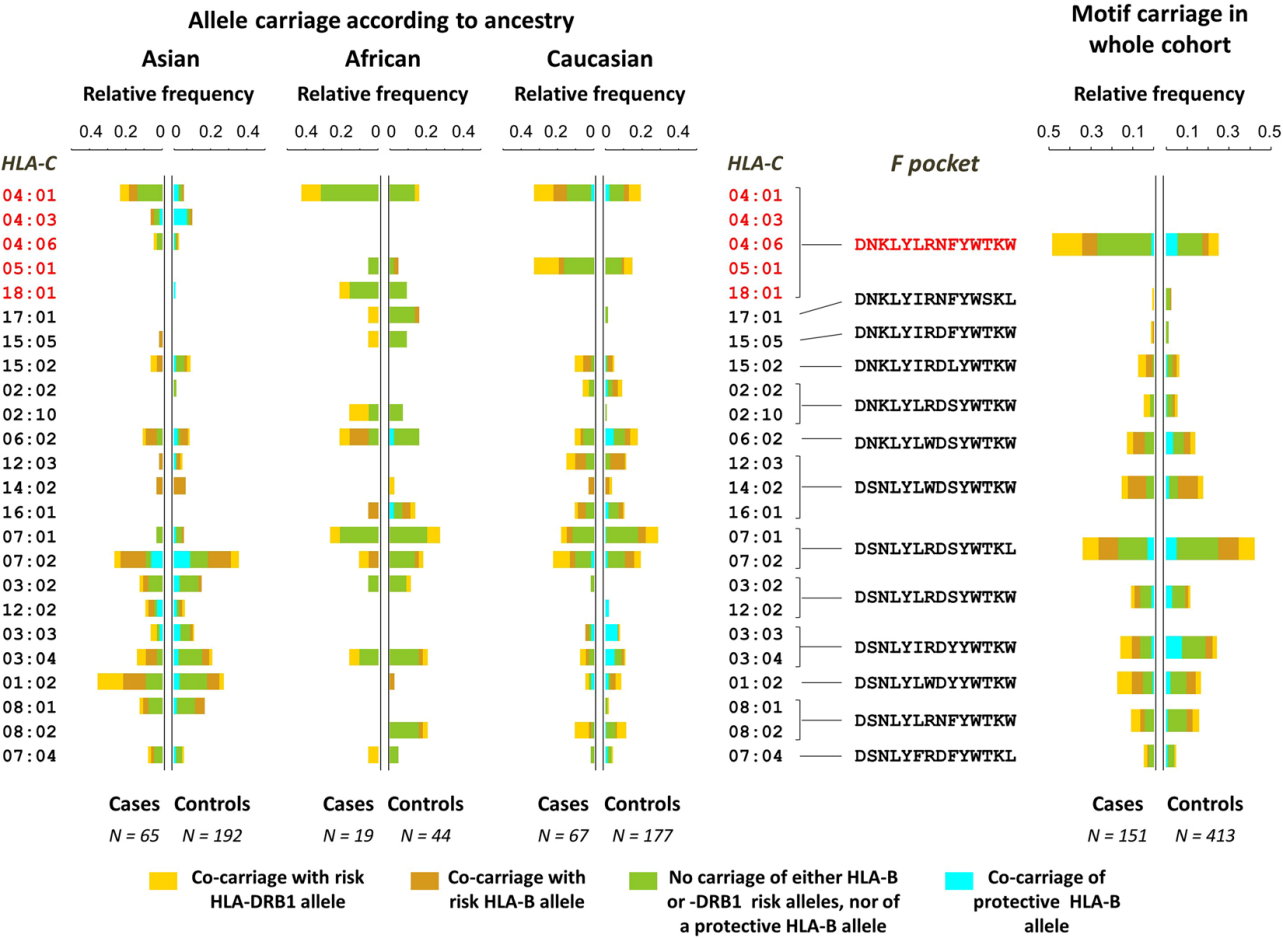
Relative frequency distributions for carriage of HLA-C alleles and characteristic F pocket motifs and co-carriage with other HLA risk or protective alleles. Results show the proportions of carriers amongst cases and controls for the *HLA-C* F pocket motifs prevalent in this cohort (N ≥ 5 carriers), and the corresponding relative frequency profiles for the alleles sharing each motif according to ancestral group. The primary risk cluster and characteristic motif are labelled in red.

Multivariate modelling was used to examine overall risk for cutaneous NVP HSR associated with the identified predisposing and protective clusters in both whole-cohort and ancestry-specific analyses. The cluster effects remain strong when the models additionally account for the independent impact of NVP metabolism as conferred by *CYP2B6* genotype, noted previously in this cohort^21^ (Table 2). Moreover, striking similarities in observed relative odds are evident across the ancestral groups (Fig. 3), despite varying allele frequency distributions (Fig. 4). Risk *HLA-B* and *HLA-DRB1* alleles are shared across several *HLA-C* allele groups in addition to the HLA-C*04:01 F pocket risk group and there is little support for any dominant haplotypic effect in cutaneous NVP HSR risk with the exception of HLA-B*35:05 carried with HLA-C*04:01 in Asians which display strong linkage disequilibrium.

**Table 2:** Multivariate logistic regression model of predisposition to cutaneous NVP HSR, according to ancestry.

	ALL OR [95% CI]	Caucasian OR [95% CI]	Asian OR [95% CI]	African OR [95% CI]
HLA-C*04:01	4.06 [2.39–6.88]	3.14 [1.49–6.62]	7.30 [2.74–19.5]	5.60 [1.22–25.75]
Other risk F pocket (DNKLYLRNFYWTKW) HLA-C*04:(03/06/07), -C*05:(01/09), -C*18:01	2.91 [1.62–5.23]	3.92 [1.74–8.86]	1.64 [0.53–5.12]	3.18 [0.53–19.04]
Risk B07 alleles HLA-B*35:05, -B*39:10, -B*51:(*01*/02), -B*54:01, HLA-B*55:(*01*/02), -B*56:(01/04), -B*67:01, HLA-B*78:01	2.32 [1.42–3.79]	2.27 [1.06–4.85]	2.19 [1.10–4.35]	2.93 [0.23–37.10]
Risk non-B07 HLA-B alleles HLA-B*13:02, B*38:(01/*02*), -B*39:(*01*/05/06/09), B*51:07	1.76 [1.02–3.02]	1.87 [0.76–4.57]	1.50 [0.72–3.12]	5.54 [0.30–101.20]
Protective HLA-B alleles HLA-B*15:(01/12/24/25/27/32/35), -B*52:01	0.18 [0.07–0.46]	0.25 [0.06–1.15]	0.18 [0.05–0.61]	
Risk HLA-DRB1 alleles HLA-DRB1*01:(*01*/02/03), -DRB1*04*:*(*04*/05/08/10)	2.00 [1.23–3.24]	1.34 [0.68–2.64]	3.23 [1.40–7.43]	4.77 [0.95–24.04]
Slow CYP2B6 metabolizer	2.07 [1.12–3.82]	3.19 [1.04–9.78]	1.66 [0.64–4.32]	1.81 [0.45–7.33]

All models have been adjusted for ethnicity as appropriate.

## Discussion

NVP HSR has been associated with multiple HLA class I and II alleles across different ethnicities. Here, utilization of high resolution typing for the cohort of HIV-1-infected patients in this study was combined with a detailed analysis of peptide binding groove properties. The analyses revealed that, despite marked variation in the observed HLA allele repertoire across the representative ethnicities, the alleles associated with cutaneous NVP HSR share the structure of specific binding pockets within the antigen-binding groove. Consideration of binding pocket structure has previously been useful for the identification of key HLA molecule risk positions in the pathology of several autoimmune diseases with HLA class I and class II allele associations as well as HIV-1 disease progression^41, 43–45^. While certain drug HSR syndromes show clear associations with only one specific allele, such as abacavir with HLA-B*57:01, such single allele associations with 100% negative predictive values are the exception rather than the rule; hence the approach described provides a potential means for exploring more complex drug HSRs or immune-based pathologies with multiple risk HLA alleles such as is observed for cutaneous NVP HSR.

### HLA class I risk allele model

Both HLA-C*04 and HLA-B*35 have been linked with cutaneous NVP HSR symptoms of varying severity in other studies^19, 21, 22, 46–48^, but with HLA-B*35/-C*04 carried as a common haplotype it has been difficult to disentangle the relative contributions of the individual alleles. Our data suggest that HLA-B*35:05 and HLA-C*04:01 may have a synergistic effect in South East Asians, but any apparent predisposition conferred by other HLA-B*35 alleles is abrogated when co-carriage of a risk *HLA-C* allele is considered. Moreover, here we demonstrate that the observed association with HLA-C*04 across ethnicities is primarily driven by the unique F pocket motif that HLA-C*04:01 shares with HLA-C*05:01 and HLA-C*18:01 which have dominant effects observed in the Hispanic and African subgroups, respectively. By focussing on an underlying biological model, this targeted analysis has therefore enabled both the confirmation of previous findings and identification of novel, less common, alleles that may be missed in exploratory analyses which require multiple comparisons to be appropriately accommodated.

Examination of the crystal structure of HLA-C*04:01 complexed with a nonameric consensus peptide (QYDDAVYKL)^31^ yields some insight into a possible mechanism for heightened predisposition to NVP HSR. The solved structure shows that Arg156, which is characteristic of all the observed HLA-C risk alleles, forms stabilising hydrogen bonds with the central portion of the peptide (QY**D**D**AV**YKL). Our docking models indicate the F pocket is a preferred binding site for NVP to interact directly with the binding groove of HLA-C. Our data therefore supports a model of cutaneous NVP HSR whereby the chemistry of the F pocket in the antigen-binding groove of the primary *HLA-C* risk molecules enables binding of NVP in the same area as the C-terminal binding of disease causing peptide ligands presented to pathogenic T cells. We propose that these peptides are anchored in the F pocket together with NVP, and hence the central portion of the peptide (P3-P5-P6) is stabilised by Arg156, and when presented together with NVP, propagate T-cell mediated responses in NVP HSR individuals. This is in keeping with other models of drug hypersensitivity, such as abacavir hypersensitivity syndrome where both drug and peptide are able to occupy the peptide binding groove of HLA-B*57:01^8, 9^. Furthermore, our proposed model is consistent with mitigation of risk being associated with diminished cell surface expression of predisposing HLA molecules, with risk HLA-C alleles being amongst the more highly expressed HLA-C molecules. Similarly, protection afforded by the protective HLA-B*15:01/-B*52:01 cluster could be explained by dominance of the more highly expressed HLA-B molecules. In the case of protective HLA-B alleles the particular HLA-B-drug—peptide combination may mimic a self-peptide-HLA combination that is tolerated by the host.

### HLA-DRB1 risk allele model

A HLA-DRB1 P4 pocket of the peptide binding groove common to the alleles HLA-DRB1*01:(*01*/02/03) and -DRB1*04*:*(*04*/05/08/10) shows a significant secondary association with cutaneous NVP HSR. Despite this, peptide elution and binding studies with a cells expressing HLA-DRB1*01:01 together with molecular modelling did not show evidence of NVP binding to HLA-DRB1*01:01 in the presence of peptide, or any influence of NVP on the repertoire of peptides presented by HLA-DRB1*01:01. It is possible that NVP has off-target or tissue specific effects on peptides *in vivo* that are not detected in the HLA-DRB1*01:01 elutions, or that the drug directly impacts TCR binding.

The P4 pocket of HLA-DRB1*01:01 and other *HLA-DRB1* risk alleles that are associated with cutaneous NVP HSR differ from the protective alleles HLA-DRB1*04:01 and -DRB1*04:15 by a single amino acid residue β71, where R(Arg) is present in risk alleles and K(Lys) is protective. Previous crystal structures for HLA-DRB1*01:01 and -DRB1*04:01 bound to TCR (HA1.7) and peptide (HA antigen from influenza A virus), have shown that K(Lys)71 has a shorter side chain and pulls the bound peptide further into the HLA groove compared to the longer sidechain of R(Arg)71 that binds the peptide to the groove in more horizontal orientation^49^. In this model, cross-reactive TCR are able to tolerate these differences, while other specific TCRs are sensitive to such differences. A similar effect on peptide orientation for positon β71 is observed in HLA-DRB1*15:01^50^. Therefore, β71 in the base of the HLA-DRB1 binding groove P4 pocket, which is significant in cutaneous NVP HSR, influences T cell recognition through its impact on the peptide preference and orientation^50–52^. Similarly in several autoimmune conditions including Graves’ disease, type I diabetes and rheumatoid arthritis, the P4 pocket positions 13, 70 and 71 separate risk and protective *HLA-DRB1* alleles^38–42, 53, 54^.

### Additional risk factors

The risk HLA alleles identified in this study appear to be required for the development of cutaneous NVP HSR, however, similar to the HLA associations with other drugs, not all individuals with the risk alleles develop hypersensitivity to NVP and additional factors also contribute to this “positive predictive value gap” (Fig. 4). For example, slow metabolizer genotypes for *CYP2B6* (516 G → T and 983 T → C) have previously been shown to correlate with both increased plasma levels of NVP and increased risk for class I HLA restricted cutaneous NVP HSR^54–56^. Alternative drug metabolism pathways may also impact predisposition to NVP HSR, such as other CYP enzymes (*CYP3A4*, *CYP2D6*, *CYP2C19 and CYP2A6*), which contribute to formation of the 12-sulfoxyl-NVP metabolite^57^ which binds selectively to histidine and cysteine residues in proteins *in vitro*. The same adducts are detected in human serum albumin isolated from the blood of NVP-treated patients^58^ as are adducts of hemoglobin with modified N -terminal valine residues^59^. This suggests pro-haptenation as another potential mechanism for HLA specific associations with hypersensitivity to NVP and may explain in part, why no shift in peptide repertoire is seen in elution studies with HLA-DRB1*01:01 cell lines after NVP treatment *in-vitro*. Additional factors that contribute to T-cell recognition of foreign antigens such as tissue and individual variation in peptides, alternative peptide processing pathways or the available TCR repertoire of an individual may also contribute to the development of and specific clinical phenotype of NVP hypersensitivity for individuals with risk HLA alleles.

In summary, this study has considered how peptide binding chemistry of the HLA antigen binding groove impacts cutaneous NVP HSR and has identified the unique F pocket conformation that defines a primary risk cluster of *HLA-C* alleles. The secondary protective and risk effects also identified in *HLA-B* and *HLA-DRB1* highlight the complexity of this particular drug HSR. Our findings suggest possible models of cutaneous NVP HSR and reveal key HLA alleles in NVP HSR risk and protection. These insights can be utilized to further investigate the nature of the bound peptide in the presence or absence of NVP and the T cell response to drug. The novel approach presented here is likely to prove useful for the study of complex HLA associations in other drug HSR syndromes and autoimmune conditions.

## Methods

### Patient Samples

Data and specimens used for this study were from a case-control analysis of NVP HSR (ClinicalTrials.gov; NCT00310843) where IRB approval had been obtained for HLA typing and the samples had previously been examined for low-resolution (two digit) HLA associations with NVP hypersensitivity^21^. The present study focused on high-resolution (four digit) typing using deep sequencing technologies. Ethics approval for this study was provided by Vanderbilt University (IRB#111684) and Murdoch University (HREC#2012/163). All methods were performed in accordance with the Australian National Health and Medical research Council (NHMRC) “*National Statement on ethical conduct in human research 2007”*.

### Patient Inclusion/Exclusion criteria

Cases (N = 151) and controls (N = 413) were at least 18 years of age, HIV-1-infected, and had previously initiated NVP-containing therapy. Cases had experienced severe cutaneous toxicity (grade 3 or 4) categorized by National Institute of Allergy and Infectious Disease (NIAID) Division of AIDS criteria. Potential cases and controls were excluded for: fewer than 150 CD4 T cells/μl within six months before initiating NVP or use of immunomodulatory medications within the first eight weeks of NVP therapy. Potential controls were excluded for: development of grade ≥1 rash within 18 weeks of initiating nevirapine or any cutaneous condition potentially attributable to nevirapine; or any systemic reaction (e.g. flu-like symptoms, arthralgia, myalgia, or conjunctivitis) attributable to nevirapine during the first 18 weeks of treatment. Further case/control specific exclusion criteria are described in the original study^19^. All participants provided written informed consent.

In this analysis, clinical notes were re-assessed independently and only cases having a primary cutaneous phenotype were included. This analysis was restricted to individuals of five distinct self-reported ethnicities and Asian, African or Caucasian ancestry was ascribed accordingly (Asian: 54 cases/209 total South-East Asian and 11/48 Taiwanese; African: 19/63 African American; Caucasian: 42/158 European and 25/86 Hispanic). Samples from the original cohort were excluded in the re-analysis for the following reasons: no sample available for HLA typing, no clinical data, sample identity issues, or race/ethnicity other than described above.

### HLA typing

Specific HLA loci were PCR amplified using sample specific MID-tagged primers that amplify polymorphic exons from HLA class I (-A, -B, -C exons 2 and 3) and class II (-DRB1, exon 2). Amplified DNA products from unique MID tagged products (up to 48 MIDs) were then pooled in equimolar ratios and subjected to library preparation, quantitation and emulsion PCR suitable for entry into the 454 FLX sequencing pipeline. Clonally enriched beads were used for 454 Titanium chemistry based sequencing on the 454 FLX+ sequencer. Sequences were then separated by MID tags and alleles called using an in-house accredited HLA allele caller software pipeline using the latest IMGT HLA allele database as the allele reference library.

### Data Analysis

Logistic regression analyses were undertaken to systematically examine differential effects on NVP HSR risk of HLA class I/II alleles and allele clusters based on HLA supertypes^4, 25^, binding pocket structure and peptide binding groups assigned from *MHCcluster* binding specificities^26, 27^. Both whole-cohort analyses and those restricted to ancestral groups were conducted and adjusted for ethnicity as appropriate. Odds ratios represent the estimated odds of HSR development amongst individuals carrying the designated allele/cluster relative to non-carriers (having all other demographic variables the same), and have been calculated as exponentiated model coefficients with the corresponding Wald confidence limits calculated similarly. P-values have also been derived from Wald tests of model coefficients.

First pass class I HLA binding groove B and F pocket positions were as defined in Sidney *et al*.^4^. Further HLA class I analysis included binding pockets A and C-E^5^. E and F pocket positions are overlapping at positions 97, 114 and 147^4, 5^, thus for additional E/F pocket analysis all E and F pocket positions were included^4, 5^. HLA class II pockets were as previously defined^7^. Expected levels of HLA-C cell surface expression were calculated as the sum of two allelic median fluorescence intensity (MFI) coefficients among cases and controls as previously assigned^28–30^.

### Molecular docking

The crystal structure of HLA molecules (HLA-C*04:01 (Protein Data Bank; PDB 1QQD); HLA-DRB1*01:01, (PDB 1FYT); HLA-B*15:01 (PDB 1XR8)) were utilised with AutoDock Vina for molecular docking predictions between NVP and the HLA alleles of interest. For modelling other HLA alleles, amino acid sequences were taken from IMGT HLA (http://www.ebi.ac.uk/ipd/imgt/hla/allele.html). The HLA structures were generated based on the most similar solved structure in the PDB, using a swiss-model (http://swissmodel.expasy.org/). DOCKER was used to align the HLA sequences (PILEUP, GCG Wisconsin Package), calculate sequence similarity based on a Blosum62 matrix, and output values for each protein position to correspond to atomic coordinates, which were plotted in 3-dimensions using PyMol (The PyMOL Molecular Graphics System, Version 1.8 Schrödinger, LLC.).

### Peptide Elutions using Single Antigen Lines

LG2 cells homozygously expressing HLA-DRB1*01:01 were incubated with nevirapine (100 μg/mL) for 14 hr at 37 °C. Cell lysate was centrifuged at 100,000 × g for 1 hr and the supernatant was collected and passed through a 0.8/0.2 μm filter (VWR International, TX). The filtrate was collected and passed through a sepharose CL-4B (Sigma-Aldrich, MO) column, then passed through a column with protein A sepharose (PAS) beads (Sigma) coated with MK-D6.1 (MTCC HB-3, VA) antibody which served as an irrelevant antibody (specific for the mouse class II molecule, I-Ad) used to derive a negative control peptide extract. Next, the filtrate was passed through a second PAS column coated with L243 antibody (Biolegend) which captures HLA-DR molecules. The columns were washed and peptides eluted with 0.2 M glacial acetic acid. The eluted peptides were then collected and spun at 3,500 g at 4 °C until 98% of the solution had passed through Millipore ultrafiltration units with a 10 kDa cut-off (EMD Millipore, MA). The filtrate was then collected and vacuum-concentrated for subsequent LC-MS analysis.

### Mass Spectrometry and Peptide analysis

Dried samples were brought up in 0.1% acetic acid and directly loaded onto an in-house, packed C18 column^55, 56^. Briefly, an irregular C18 (5–20 μm diameter) capillary precolumn (360 μm outer diameter, 75 μm inner diameter) was connected to a C18 (5 μm diameter) analytical capillary column (360 μm outer diameter, 50 μm inner diameter) equipped with an electrospray emitter tip. Peptides were eluted by a 90 min 0–60% B gradient (A: 0.1 M acetic acid; B: 70% ACN, 0.1 M acetic acid) using an Agilent 1100 HPLC at a flow rate of 60 nL/min. The RP-HPLC elution was electrospray-ionized into an Orbitrap Fusion Tribrid mass spectrometer (Thermo Scientific). Data analysis was performed using Xcalibur software (Thermo Scientific). Raw data files were searched against the RefSeq database using OMSSA^57^. MS2 searches used the following parameters: no enzyme specificity, e-value cutoff of 1, and a variable modification of methionine oxidation. Mass tolerances for intact and product ion masses were set at ± 0.1 Da and ± 0.35 Da, respectively. Additionally, MS2 data was searched using either c- and z·- fragments (ETD) or b-and y- fragments (CAD). All peptide hits were subject to manual interpretation of MS2 spectra.

### MHC-Peptide Binding Assays

Assays to quantitatively measure peptide binding to HLA-DRB1*01:01 (class II) MHC molecules are based on the inhibition of binding of a high affinity radiolabeled peptide to purified MHC molecules, and were performed essentially as described elsewhere^58, 59^. In brief, 0.1–1 nM of radiolabeled peptide was co-incubated at room temperature with 1 nM to 1 µM of purified HLA-DRB1*01:01 MHC in the presence of a cocktail of protease inhibitors and 4 mg/mL of nevirapine in DMSO vs. DMSO alone, respectively. Following a two day incubation, MHC bound radioactivity was determined by capturing the MHC/peptide complexes on L243 (anti HLA-DR) antibody coated Lumitrac 600 plates (Greiner Bio-one, Frickenhausen, Germany), and measuring bound cpm using the TopCount (Packard Instrument Co., Meriden, CT) microscintillation counter. In the case of competitive assays, the concentration of peptide yielding 50% inhibition of the binding of the radiolabeled peptide was calculated. Under the conditions utilized, where [label] < [MHC] and IC50 ≥ [MHC], the measured IC50 values are reasonable approximations of the true Kd values^60, 61^. Each competitor peptide was tested at six concentrations covering a 100,000-fold dose range. As a positive control, the unlabeled version of the radiolabeled probe was also tested in each experiment.

### Data Availability

The datasets generated during and/or analysed during the current study are available from the corresponding author on reasonable request.

### URLS

MHC cLuster NetMHCpan-2.8 (http://www.cbs.dtu.dk/services/MHCcluster/), NetMHCII Server (http://www.cbs.dtu.dk/services/NetMHCII/), IPD-IMGT/HLA (https://www.ebi.ac.uk/ipd/imgt/hla/), Protein Data bank (PDB) (http://www.rcsb.org/pdb/home/home.do).

## Electronic supplementary material

Supplementary Tables and Figures
