# Supplementary material for: Shared peptide binding of HLA Class I and II alleles associate with cutaneous nevirapine hypersensitivity and identify novel risk alleles
Source: Sci Rep. 2017 Aug 17;7:8653. doi: 10.1038/s41598-017-08876-0 (PMC5561238; doi:10.1038/s41598-017-08876-0)
Supplement: Supplementary file 1 — Supplementary Tables and Figures [file 41598_2017_8876_MOESM1_ESM.pdf]

Shared peptide binding of HLA Class I and II alleles associate with cutaneous nevirapine hypersensitivity and identify novel risk alleles

Rebecca Pavlos[1¶], Elizabeth J. McKinnon[1¶], David A. Ostrov[2], Bjoern Peters[3], Soren Buus[4], David Koelle[5,6,7,8,9], Abha Chopra[1], Ryan Schutte[2], Craig Rive[1], Alec Redwood[1], Susana Restrepo[2], Austin Bracey[2], Thomas Kaever[3], Paisley Myers[10], Ellen Speers[10], Stacy A.Malaker[10], Jeffrey Shabanowitz[10], Jing, Yuan[11], Silvana Gaudieri[1,12,13], Donald F. Hunt[10], Mary Carrington[14,15,16], David W. Haas[13, 17], Simon Mallal[1,13], Elizabeth J. Phillips[1,13]*

Supplemental Information

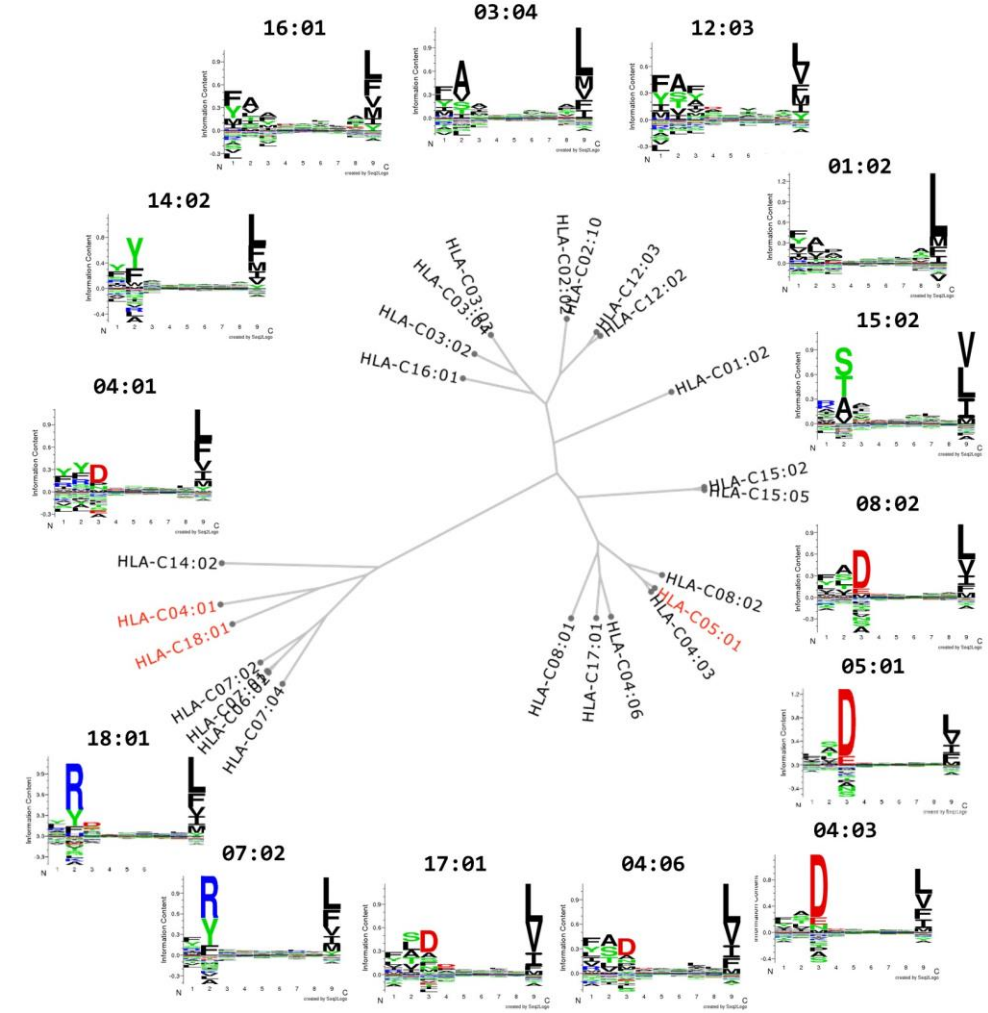

Figure S1. HLA-C *MHCcluster* analysis. The distance tree shows sequence logos for predicted peptide binding sequences. Principal *HLA-C* risk alleles for NVP HSR rash (*HLA-C*04:01, -05:01 and -18:01*) are shown in red font.

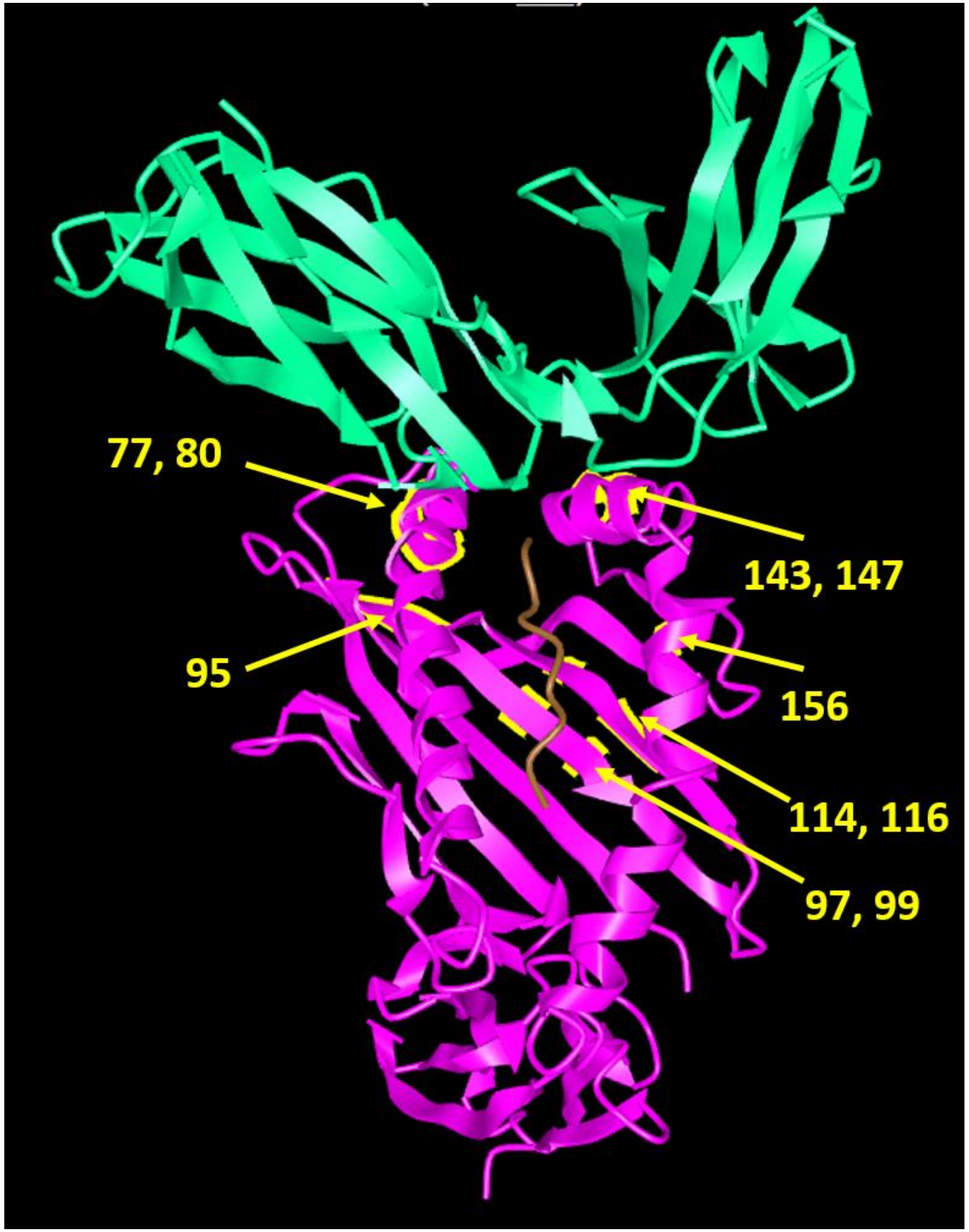

Figure S2. Crystal Structure of KIR2DL2 bound to HLA-Cw4 with key cutaneous NVP HSR risk allele positions hihglighted. Structure PDB ID:1IM9 is shown[67] . KIR2DL2 is green, HLA-Cw4 is magenta and the bound peptide is brown. Key positions from the F pocket, unique in HLA-C risk alleles are highlighted in yellow. Position 77 and 80 are KIR contact resdiues, and positions 143 and 147 also interact closely with KIR. Positions 95, 97, 99, 114,and 116 lie on the base of the peptide binding groove and normally interact with the bound peptide. Position 156 is located on the α2 helix and can stabilise postions P3, P5 and P6 of the bound peptide.

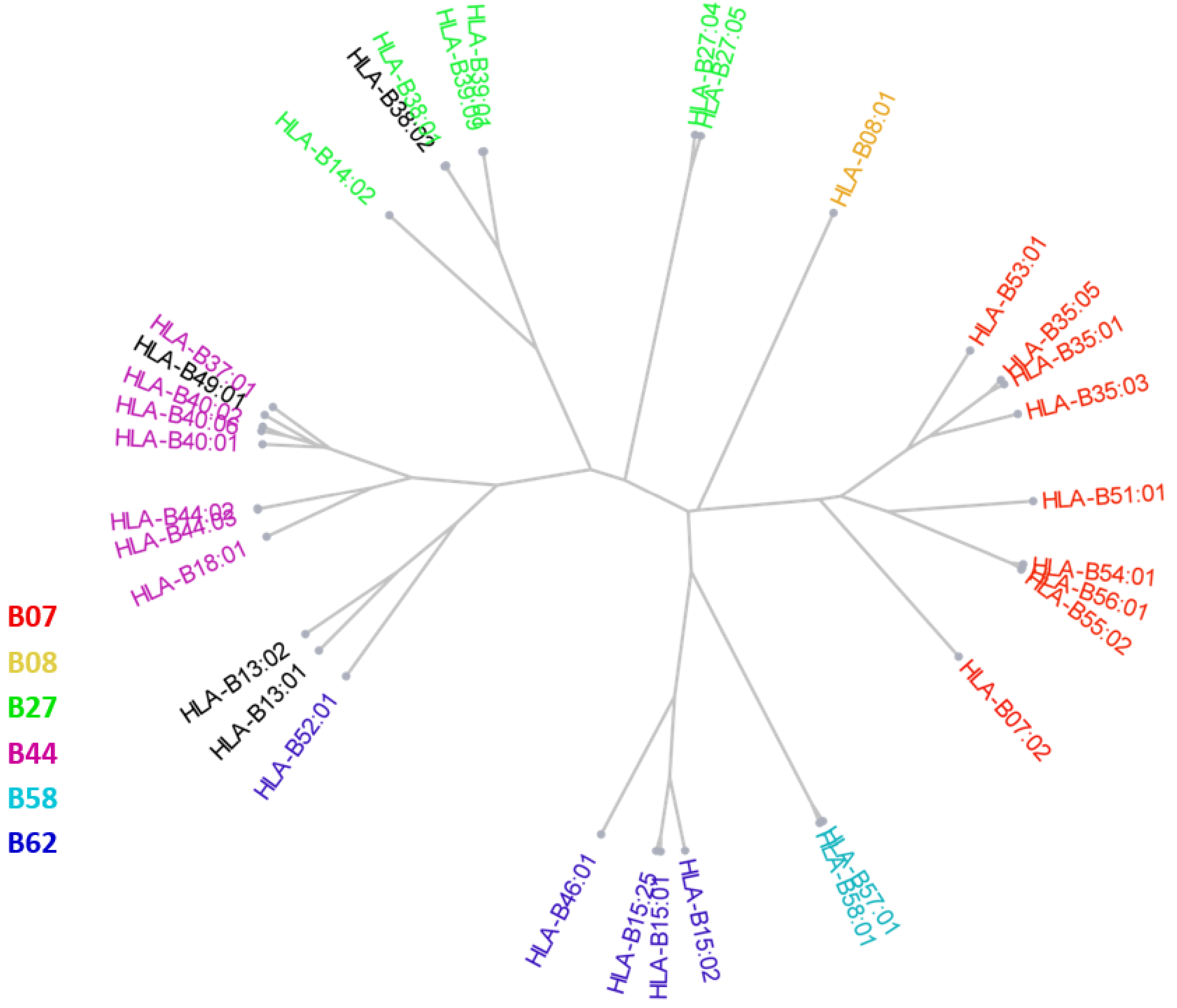

Figure S3. **HLA-B *MHCcluster* tree based on predicted peptide binding for HLA-B**. Alleles prevalent in this cohort (N≥10 carriers). HLA-B supertypes are colour coded as shown in the key. Alleles which are not classified by supertypes are in black font.

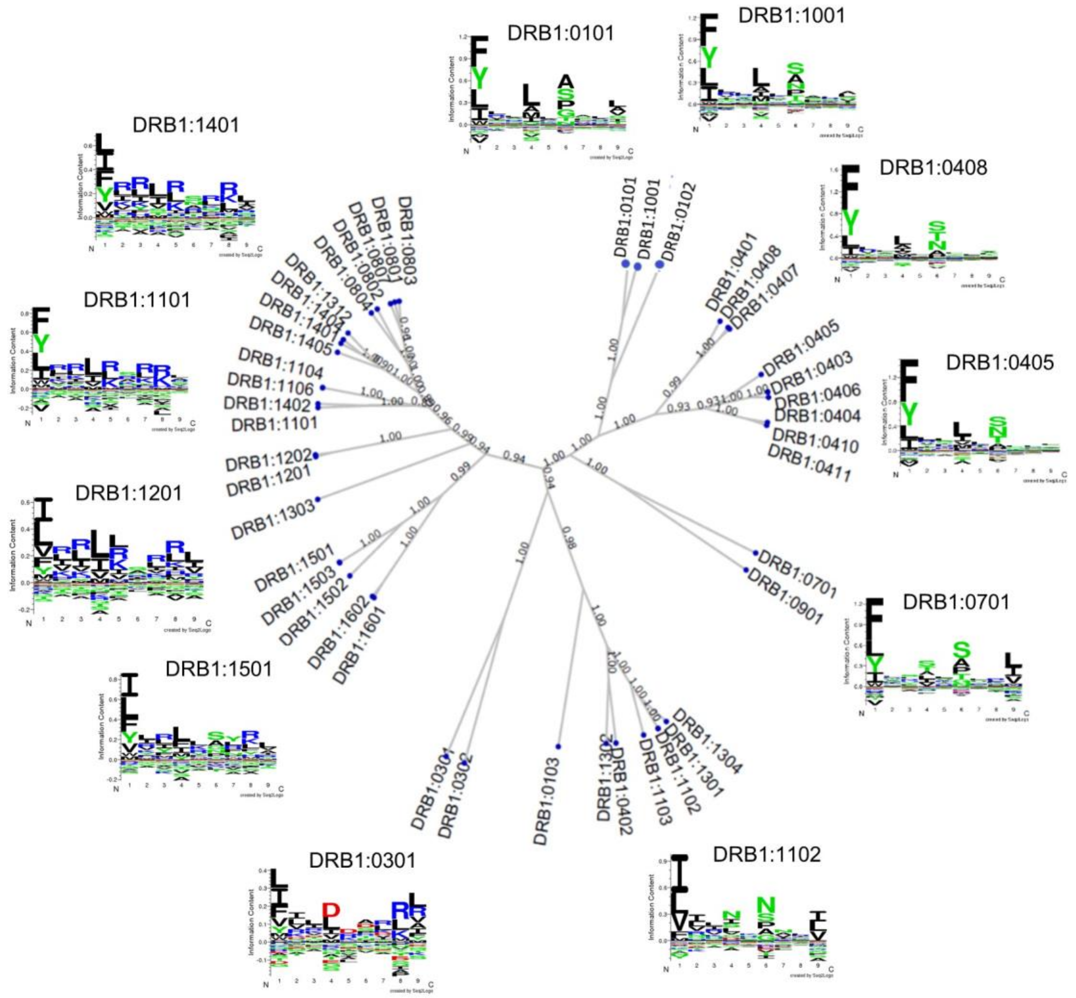

Figure S4. *MHCcluster* analysis for HLA-DRB1 alleles groups HLA-DRB1 risk alleles for cutaneous NVP HSR. The distance tree shows sequence logos for predicted peptide binding sequences.

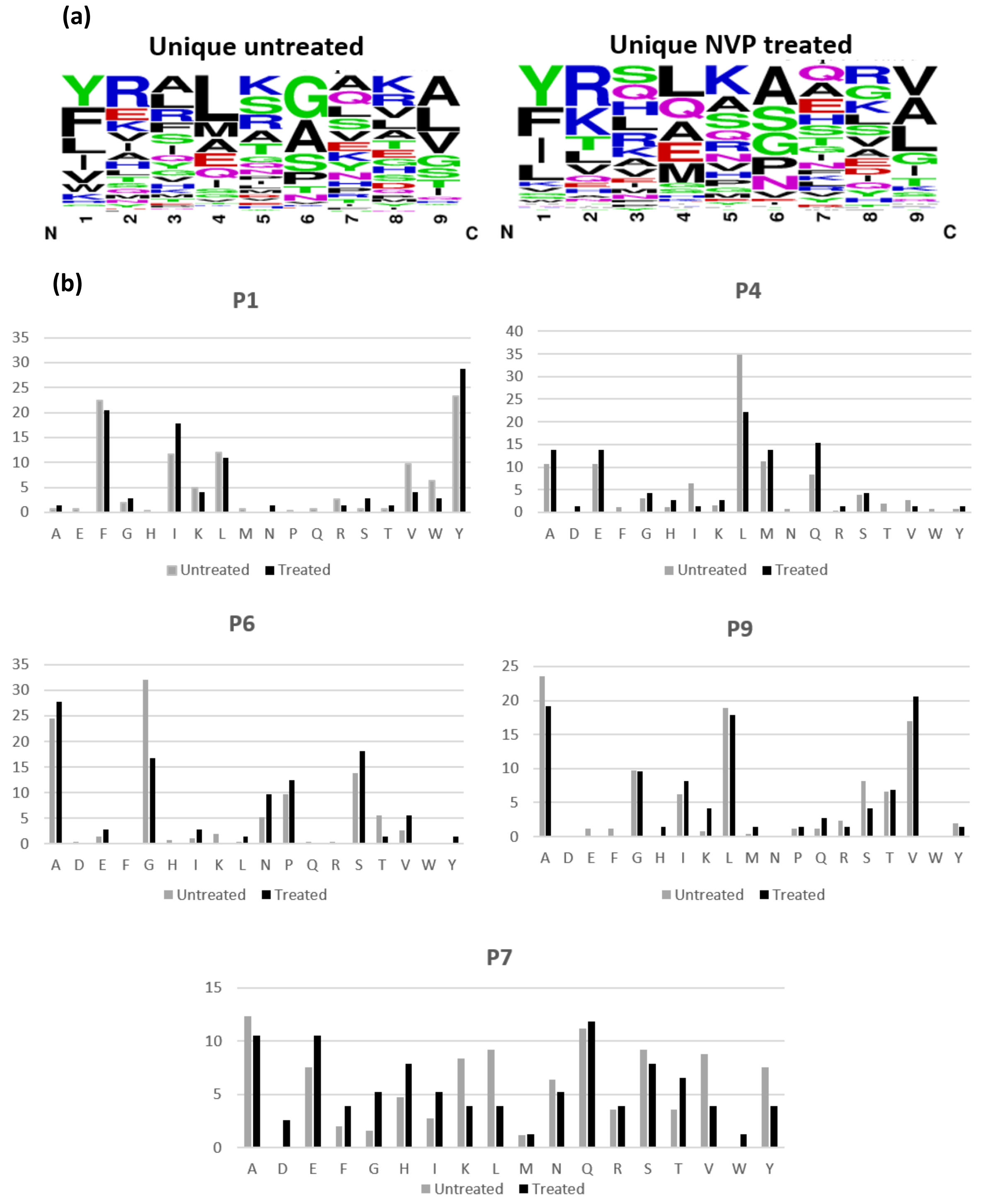

Figure S5. **HLA-DRB1*01:01 elutions in the presence and absence of NVP treatement. (a)** *Weblogo* of amino acid frequencies for unique untreated versus unqiue nevirapine treated peptide elution sets**. (b)** Frequency plots for amino acids observed at each anchor residue of the peptide P1, P4, P6 and P9, and P7 predicted to interact with the P4 pocket, for the HLA-DRB1*01:01 elutions in unique NVP treated (black) and NVP untreated peptides (gray).

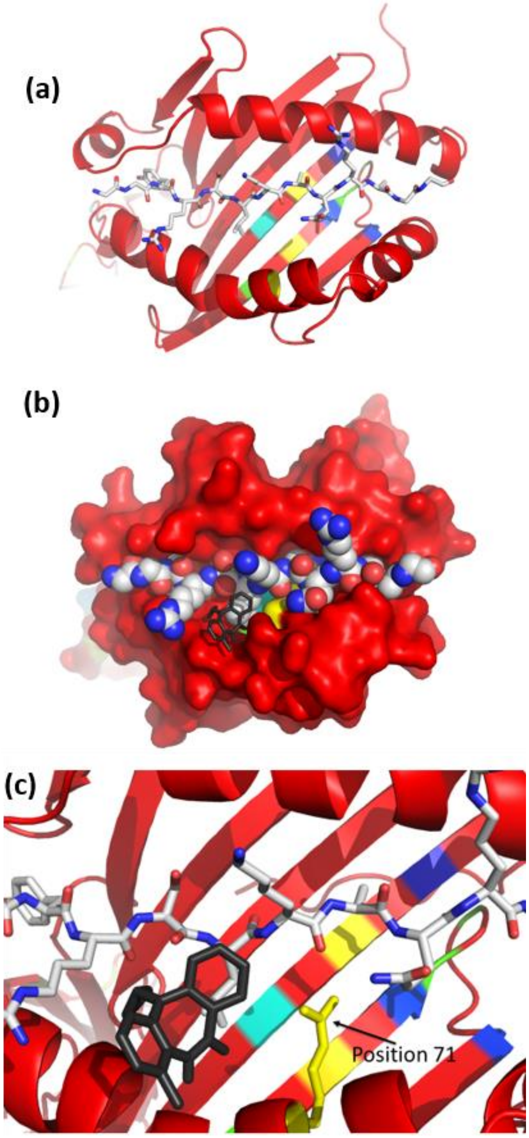

Figure S6. Protective and risk HLA-DR molecules differ in sites that influence peptide binding. The crystal structure of risk allele HLA-DRB1*01:01, from PDB 1FYT, is shown colored by sequence similarity (Blosum62 matrix) to protective allele HLA-DRB1*04:01. Blosum62 similarity values are: blue, 40–50, cyan, 50–60, green, 60–70, yellow, 70–80, orange 80-90, and red 90–100. Position β71 is coloured yellow. The sequence motif identified from peptides bound to HLA-DRB1*01:01 in NVP treated cells, YRSLKAQRV, is shown in the antigen binding cleft as sticks **(a)** and **(c)** spheres in panel **(b).** Molecular docking predicts that NVP (shown as black sticks) does not bind within the antigen binding cleft of HLA-DRB1:01 as shown in panels **(b)** and **(c).**

No peptide		With poly-glycine peptide	
**B pocket (kcal/mol)**	**F pocket (kcal/mol)**	**B pocket (kcal/mol)**	**F pocket (kcal/mol)**
-8.0	-8.1	-5.2	-7.5

Table S1. Molecular docking prediction of binding interactions between NVP and the B or F pockets of HLA-C*04:01 in the presence and absence of poly-glycine peptide. AutoDock Vina (Scripps) scores for the highest predicted poses (orientation and conformation) are shown.

	AA position B pocket									
HLA B Alleles	7	9	24	34	45	63	66	67	70	99
[#]15:(01/12/24/25/35)	Y	Y	A	V	M	E	I	S	N	Y
[#]15:27	.	.	.	.	.	.	.	.	.	F
[#]15:32	.	.	.	.	.	.	.	.	.	S
[#]52:01	.	.	.	.	T	.	.	.	.	Y
[#]15:02	.	.	.	.	M	N	.	.	.	Y
[&]15:11	.	.	.	.	M	N	.	Y	.	Y
15:03	.	.	S	.	E	.	.	.	.	Y
15:(18/23/10)	.	.	S	.	E	N	.	C	.	Y
[&]13:(01/02)	.	.	T	.	M	.	.	.	.	Y
[&]35:19	.	.	.	.	K	N	.	F	.	Y
[&]35:20, [&]51:07	.	.	.	.	T	N	.	.	.	Y
53:01,78:01,51:(01/02/04/08),35:(01/02/03/04/05/08/43)	.	.	.	.	T	N	.	F	.	Y
54:01	.	.	.	.	G	N	.	Y	Q	Y
55:(01/02),56:(01/03/04)	.	.	.	.	E	N	.	Y	Q	Y
57:(01/02/03/04)	.	.	.	.	M	.	N	M	S	Y
58:(01/02)	.	.	.	.	T	.	N	M	S	Y
[#]46:01	.	.	.	.	M	.	K	Y	Q	Y
15:(16/17)	.	.	.	.	M	.	N	M	S	Y
40:(01/02/06/16/74),41:(01/02),44:15,45:01,[&]49:01,50:(01/02)	.	H	T	.	K	.	.	.	.	Y
44(:02/03/05/07)	.	.	T	.	K	.	.	.	.	Y
[&]47:01	.	.	T	.	K	.	.	.	.	F
27:(02/03/04/05/06)	.	H	T	.	E	.	.	C	K	Y
18:(01/02)	.	H	S	.	T	N	.	.	.	Y
37:(01/13)	.	H	S	.	T	.	.	.	.	S
48:(01/03)	.	.	S	.	E	.	.	.	.	Y
[&]42:02	.	H	S	.	E	N	.	Y	Q	Y
39:09	.	.	S	.	E	N	.	C	.	S
39:10	.	.	S	.	E	N	.	Y	.	Y
07:(02/05),4201,67:01,81:01	.	.	S	.	E	N	.	Y	Q	Y
14:(01/02/03),38:(01,02),39:(01/05/06/14/15)	.	.	S	.	E	N	.	C	.	Y
08:01	.	D	S	.	E	N	.	F	.	Y
[&]82:02	.	.	S	.	E	N	.	Y	Q	F

Table S2. Protective HLA-B alleles in cutaneous NVP HSR have a shared B pocket sequence. The HLA-B alleles protective for cutaneous NVP HSR are on a black background. Yellow amino acids differ between protective HLA-B alleles. Red amino acids are conserved within the protective HLA-B allele group and show variation amongst other HLA-B alleles in the cohort. [#]B62 supertype. [&]Unclassified supertype.

	AA position															
	74	77	80	81	84	95	97	114	116	123	133	143	146	147	152	156
F pocket[6]																
E pocket[5]																
F pocket[5]																
HLA-C risk E/F pocket sequence	D	N	K	L	Y	L	R	N	F	Y	W	T	K	W	E	R*
HLA-B risk alleles group 1						L	R	N	F/L						V	L
39:01	D	S	N	L	.	L	R	N	F	.	.	.	.	.	V	L
67:01	D	S	N	L	.	L	R	N	F	.	.	.	.	.	V	L
39:09	D	S	N	L	.	L	R	N	F	.	.	.	.	.	V	L
39:05	Y	S	N	L	.	L	R	N	F	.	.	.	.	.	V	L
38:01	Y	N	I	A	.	L	R	N	F	.	.	.	.	.	V	L
38:02	Y	N	T	A	.	L	R	N	F	.	.	.	.	.	V	L
56:04	D	S	N	L	.	L	R	N	L	.	.	.	.	.	V	L
82:02	D	S	N	L	.	L	R	N	L	.	.	.	.	.	V	D
HLA-B risk alleles group 2						W	T	N	L/Y							L
39:06	D	S	N	L	.	W	T	N	F	.	.	.	.	.	V	L
54:01	D	S	N	L	.	W	T	N	L	.	.	.	.	.	V	L
55:01	D	S	N	L	.	W	T	N	L	.	.	.	.	.	E	L
55:02	D	S	N	L	.	W	T	N	L	.	.	.	.	.	V	L
56:01	D	S	N	L	.	W	T	N	L	.	.	.	.	.	V	L
78:01	D	S	N	L	.	W	T	N	Y	.	.	.	.	.	E	L
40:06	Y	S	N	L	.	W	T	N	Y	.	.	.	.	.	V	L
13:02	Y	N	T	A	.	W	T	N	L	.	.	.	.	.	V	L
51:01	Y	N	I	A	.	W	T	N	Y	.	.	.	.	.	E	L
51:02	Y	N	I	A	.	W	T	N	Y	.	.	.	.	.	E	L
51:07	Y	N	I	A	.	W	T	N	Y	.	.	.	.	.	E	L
51:08	Y	N	I	A	.	W	T	N	Y	.	.	.	.	.	V	D
52:01	Y	N	I	A	.	W	T	N	Y	.	.	.	.	.	E	L
35:05	Y	S	N	L	.	L	S	D	S	.	.	.	.	.	V	L

*HLA-C*04:06 carries L156.

Table S3. Risk HLA-B alleles in cutaneous NVP HSR characterised according the E/F pocket sequence. A comparison with the risk HLA-C F pocket is shown. The HLA-B risk alleles for cutaneous NVP HSR are shown in two groups based on sequence similarity at the amino acid positions 95, 97, 114, 116, 152 and 156. Variation in defined E and F pocket for HLA-B risk alleles are shown by colour code. Red = F pocket, Blue = E pocket 5. Green = F pocket residues shared by HLA-C risk alleles for cutaneous NVP HSR.

HLA-DRB1 Alleles								
HLA-DRB1 Alleles	13	70	71	73	74	76	77	78
01:(01/02/03), 04:(04/05/08/10)	F/H	Q	R	A	A	D	T	Y
04:(03/06/07/11)	H	.	.	.	E	.	.	.
04:(01/15)	H	.	K	.	.	.	.	.
10:01:01	F	R	.	.	.	.	.	.
04:34:01	P	.	K	.	.	.	.	.
14:02:01	S	.	.	.	.	.	.	.
04:02:01	H	D	E	.	.	.	.	.
15:(01/02/03/04/06)	R	.	A	.	.	.	.	.
14:21:01	S	.	K	.	.	.	.	.
09:01:01	F	R	.	.	E	.	.	V
07:01:01	Y	D	.	G	Q	.	.	V
07:15:01	Y	D	.	G	Q	.	.	.
08:(01/02/03/04/07/09)	G	D	.	.	L	.	.	.
11:(02/03), 13:(01/02/04/16)	S	D	E	.	.	.	.	.
11:(01/04/06),13:(05/12)	S	D	.	.	.	.	.	.
12:(01/02)	G	D	.	.	.	.	.	.
13:03:01	S	D	K	.	.	.	.	.
14:(01/05)	S	R	.	.	E	.	.	.
14:04:01	G	R	.	.	E	.	.	.
14:10:01	H	R	.	.	E	.	.	.
16:(01/02)	R	D	.	.	.	.	.	.
03:(01/02/81)	S	.	K	G	R	.	N	.

Table S4. Risk HLA-DRB1 alleles for cutaneous NPV HSR share P4 residues. The HLA-DRB1 risk alleles for cutaneous NVP HSR are shown on the black background. Red amino acid positions are conserved between the HLA-DRB1 risk alleles and show most variation compared to other HLA-B alleles in the cohort.
